# Physiological Induction of Regulatory Qa-1-Restricted CD8+ T Cells Triggered by Endogenous CD4+ T Cell Responses

**DOI:** 10.1371/journal.pone.0021628

**Published:** 2011-06-27

**Authors:** Aditi Varthaman, Marc Clement, Jamila Khallou-Laschet, Giulia Fornasa, Anh-Thu Gaston, Michael Dussiot, Giuseppina Caligiuri, Harvey Cantor, Srinivas Kaveri, Antonino Nicoletti

**Affiliations:** 1 UMRS698 INSERM, Univ Denis Diderot, Sorbonne Paris Cité, Paris, France; 2 UMRS872 INSERM, Centre de Recherche des Cordeliers, Equipe 16, Univ Pierre et Marie Curie, Paris, France; 3 Department of Cancer Immunology and AIDS, Dana-Farber Cancer Institute, Boston, Massachusetts, United States of America; 4 Department of Pathology, Harvard Medical School, Boston, Massachusetts, United States of America; Weizmann Institute of Science, Israel

## Abstract

T cell-dependent autoimmune diseases are characterized by the expansion of T cell clones that recognize immunodominant epitopes on the target antigen. As a consequence, for a given autoimmune disorder, pathogenic T cell clones express T cell receptors with a limited number of variable regions that define antigenic specificity. Qa-1, a MHC class I-like molecule, presents peptides from the variable region of TCRs to Qa-1-restricted CD8+ T cells. The induction of Vß-specific CD8+ T cells has been harnessed in an immunotherapeutic strategy known as the “T cell vaccination” (TCV) that comprises the injection of activated and attenuated CD4+ T cell clones so as to induce protective CD8+ T cells. We hypothesized that Qa-1-restricted CD8+ regulatory T cells could also constitute a physiologic regulatory arm of lymphocyte responses upon expansion of endogenous CD4+ T cells, in the absence of deliberate exogenous T cell vaccination. We immunized mice with two types of antigenic challenges in order to sequentially expand antigen-specific endogenous CD4+ T cells with distinct antigenic specificities but characterized by a common Vß chain in their TCR. The first immunization was performed with a non-self antigen while the second challenge was performed with a myelin-derived peptide known to drive experimental autoimmune encephalomyelitis (EAE), a mouse model of multiple sclerosis. We show that regulatory Vß-specific Qa-1-restricted CD8+ T cells induced during the first endogenous CD4+ T cell responses are able to control the expansion of subsequently mobilized pathogenic autoreactive CD4+ T cells. In conclusion, apart from the immunotherapeutic TCV, Qa-1-restricted specialized CD8+ regulatory T cells can also be induced during endogenous CD4+ T cell responses. At variance with other regulatory T cell subsets, the action of these Qa-1-restricted T cells seems to be restricted to the immediate re-activation of CD4+ T cells.

## Introduction

The regulatory potential of Qa-1-restricted CD8+ T cells has been harnessed in the immunotherapeutic strategy known as the T cell vaccination (TCV; [1]). The experimental procedure of TCV comprises the injection of activated, attenuated, T cell clones expressing one particular Vß sequence in their TCR. Such an administration leads to the expansion of regulatory suppressive CD8+ T cells that detect Vß peptides derived from the non-hypervariable region of the TCR (that is not directly involved in antigen recognition) presented by Qa-1 molecules on the vaccinating cells [2]. Once expanded these regulatory T cells can recognize and specifically target activated autoreactive T cells that express similar Vß chains. Surprisingly, the involvement of such regulatory Qa-1-restricted CD8+ T cells has never been assessed in a pathophysiological situation where a first CD4+ Vß-dominant immune response would be followed by a second CD4+ T cell response involving clones expressing a TCR of the same Vß family. In other words, can the regulatory Qa-1-restricted CD8+ T cells triggered upon a first CD4+ T cell response impede a second CD4+ T cell response if both CD4+ T cell responses mobilize clones belonging to the same Vß family?

To address this question, we used sequential antigenic immunization procedures. During the first immunization, we aimed at expanding Qa-1-restricted CD8+ T cells specific for CD4+ T cells directed against non-self antigens. We then tested whether the Qa-1-restricted CD8+ T cells induced upon the first CD4+ T cell response could control a second CD4+ T cell response implicated in the pathogenesis of murine experimental autoimmune encephalomyelitis (EAE). In our experimental design, the two sequential CD4+ T cell responses employed lymphocytes belonging to the same Vß family.

Our data show that, apart from the immunotherapeutic T cell vaccination procedure, Qa-1-restricted CD8+ T specialized regulatory T cells can also be induced by endogenous CD4+ T cell responses. Furthermore, an unforeseen level of regulation of the T cell responses is unraveled by our findings, which support the hypothesis of a continuous reshaping of the regulatory T cell repertoire dictated by previous CD4+ T cell responses.

## Materials and Methods

### Mice

Six-week old female C57BL/6 (H-2K^b^, Qa-1^b^) mice were purchased from Janvier Laboratories. Generation of Qa-1-deficient mice (H-2K^b^) has been previously described [3]. During the course of the experimentation, all mice were maintained in pathogen-free conditions and mice were handled in accordance with European Union directives (86/609/EEC) on the care and use of laboratory animals. The investigation was approved by the Animal Ethics Committee of the Institut National de la Santé et de la Recherche Médicale. The review and approval of the study was also obtained by the Local Animal Ethics Committee - Comite d'ethique Bichat/Debre' (No. B 7518 03).

### Immunization with peptide antigens

Six week-old female C57BL/6 (Janvier, France) mice were immunized with 100 µg of 50V Pigeon Cytochrome C (AEGFSYTVANKNKGIT, NeoMPS) or chicken Ovalbumin (OVA, Sigma) in emulsion with Complete Freund's Adjuvant (CFA, Sigma). A final volume of 200 µl was injected subcutaneously at four different points on the flanks. Control mice were immunized with PBS in emulsion with CFA.

### Induction of EAE

EAE was actively induced in mice upon immunization with 200 µg of MOG-35-55 peptide (MEVGWYRSPFSRVVHLYRNGK, NeoMPS) in emulsion with CFA (Sigma) 1∶1 by volume containing 800 µg of non-viable desiccated Mycobacterium tuberculosis H37RA (Difco Laboratories). A total volume of 200 µl was injected subcutaneously at 4 sites over the flanks. In addition, 300 ng of pertussis toxin (List Biological Laboratories) was given intravenously on the same day and 2 days later. The grid for the assessment of the clinical progression of EAE was adopted from Strommes et al. [4]. The grid is as follows: 0, no signs; 0.5, partially limp tail; 1, paralyzed tail; 2, hind limb paresis; 2.5, one hind limb paralyzed; 3, both hind limbs paralyzed; 3.5, weakness in forelimbs; 4, forelimbs paralyzed; 5, moribund.

### Adoptive transfer of CD8+ T cells

CD8+ T cells were negatively enriched from the draining lymph nodes of immunized mice using a CD8+ T cell enrichment kit (BD Bioscience, 558471). CD8+ T cell purity was confirmed to be more than 90%. Three or four million purified CD8+ T cells from two mice were injected retro-orbitally into one naïve recipient mouse.

### Isolation of lymphoid cells

Cells from the spleen and lymph nodes were isolated by meshing the organs through a 100 µm filter. The red blood cells in the splenocyte suspension were lysed with ACK lysis buffer. The brain and spinal cord were dissected out and meshed as above. The mononuclear cells were isolated on a 37.5% percoll (Sigma P1644) gradient and suspended in 200 µl of PBS-2% FCS.

### Antibodies and reagents

Monoclonal antibodies specific for CD4 (clone RM4-5), CD8 (clone 53–6.7) and CD3 (clone 145-2C11) at a concentration of 2 µg/ml were used in this study. MOG-specific cells were identified using MOG_38–48_-I-A^b^ tetramers at a concentration of 4 µg/ml for 20 h at 37°C. Absolute numbers of cells in 200 µl suspensions were calculated using Beckman Coulter® Flowcount fluorospheres (ref 7547053) as a standard. Flow cytometric acquisition was performed using a BD LSRII and the data were analyzed using BD Diva software.

### Statistics

Results are expressed as means ± SEM. Differences between groups were evaluated by one-way ANOVA with Fischer's post-hoc tests, or by Mann-Whitney non-parametric tests, as appropriate. Differences between groups were considered significant when the P value was <0.05. Analysis was performed using Statview 5.0 Software (SAS Institute Inc., USA).

## Results

### Primary *in vivo* T cell activation inhibits subsequent CD4+ T cell responses belonging to the same Vß family

**Figure 1:**
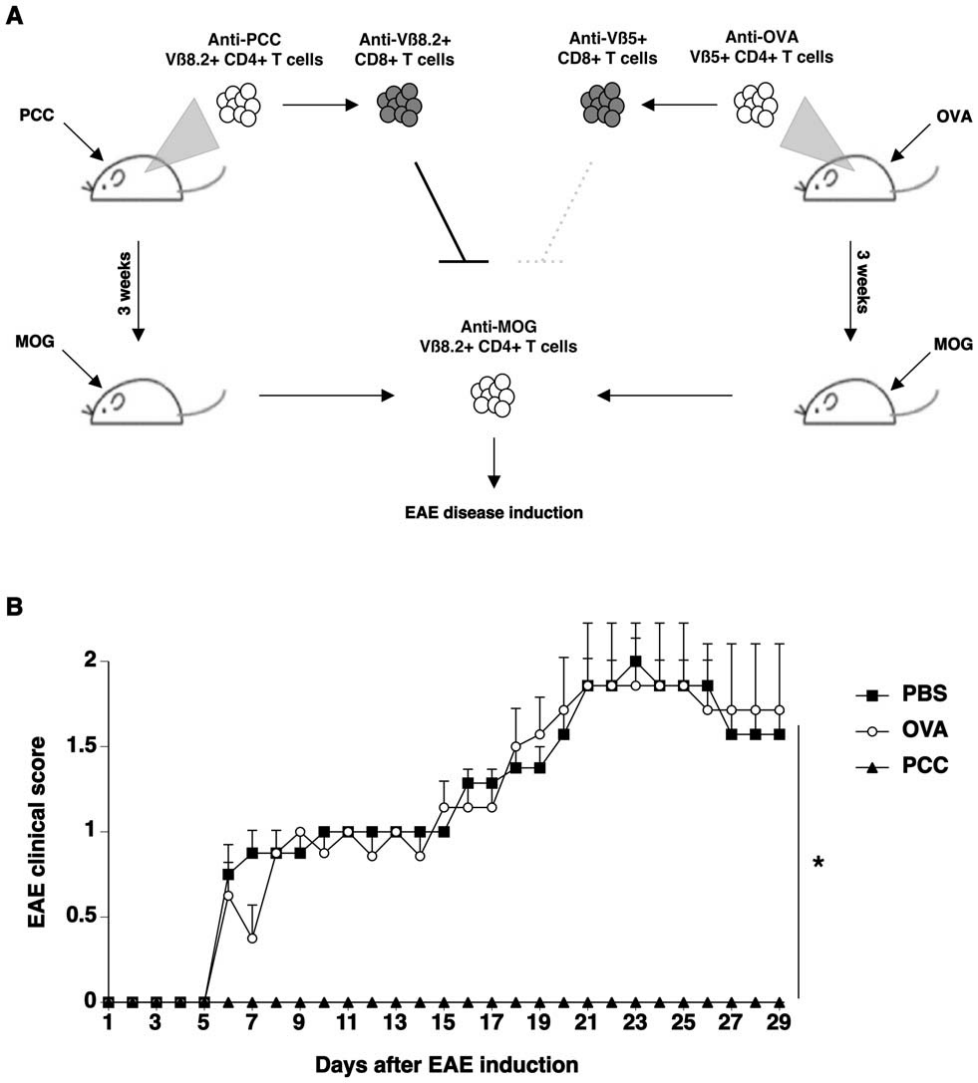
Immunization with PCC protects mice from EAE. A. Experimental design and hypothesis. C57BL/6 mice were immunized with either 50V PCC-derived peptide that induces a Vß8.2+CD4+ T cell response or an OVA-derived peptide that induces a Vß5+CD4+ T cell response. It is expected that the first T cell responses induce corresponding anti-Vß8.2 and anti-Vß5 CD8+ regulatory T cell responses. In order to test this hypothesis, mice were submitted to a second immunization with MOG. Encephalitogenic Vß8.2+ anti-MOG T cells would be controlled only by anti-Vß8.2 CD8+ T cells induced by the first antigenic challenge. In this experimental design, the control of self-reactive T cells is therefore reflected in the control of EAE development. B. Naive C57BL/6 mice were immunized subcutaneously with PBS (n = 7, squares), 100 µg of a 50V PCC-derived peptide (PCC, triangles, n = 5), or 100µg of an OVA-derived peptide (OVA, circles, n = 7) in CFA. Three weeks later, EAE was actively induced by subcutaneous immunization with MOG35-55 peptide and intravenous pertussis toxin (300 ng) injections on the day of induction and 2 days later. The clinical course of EAE was analyzed using a paralysis grading score over 30 days. Representative of 2 independent experiments. Mean ± SEM. * indicates that the p-value<0.001 as assessed using ANOVA Fisher's PLSD.

Studies on TCV have demonstrated that vaccination of mice using *ex vivo* activated and attenuated Vß8.2+CD4+ T cells protects mice from MOG-specific Vß8.2+CD4+ T cell-induced EAE [5]. We hypothesized that the endogenous activation of Vß8.2+CD4+ T cells might trigger a protective effect similar to that induced by the vaccinating T cells during TCV. Immunization with a peptide derived from the Pigeon Cytochrome C (PCC), a model non-self antigen in our studies, induces the activation of PCC-specific CD4+ T cells belonging predominantly to the Vß8.2 family [6], [7]. We thus immunized mice with either PCC or a control peptide derived from ovalbumin (OVA) that induces a Vß5+ CD4+ T cell response. Three weeks later, the mice were immunized with MOG to induce EAE. The disease course was followed by the development of paralysis. Mice that were previously immunized with PCC were completely protected from disease development. Importantly, all the mice immunized with OVA succumbed to EAE with normal kinetics of disease evolution as compared to the control mice (Figure 1). Thus, a previous activation of endogenous Vß8.2+CD4+ T cells specific for a non-self antigen - but not Vß5+CD4+ T cells - protects mice from anti-MOG Vß8.2+CD4+ T cell-mediated EAE development.

### Sustained CD8+ T cell activation is required for Vß-specific T cell suppression

**Figure 2:**
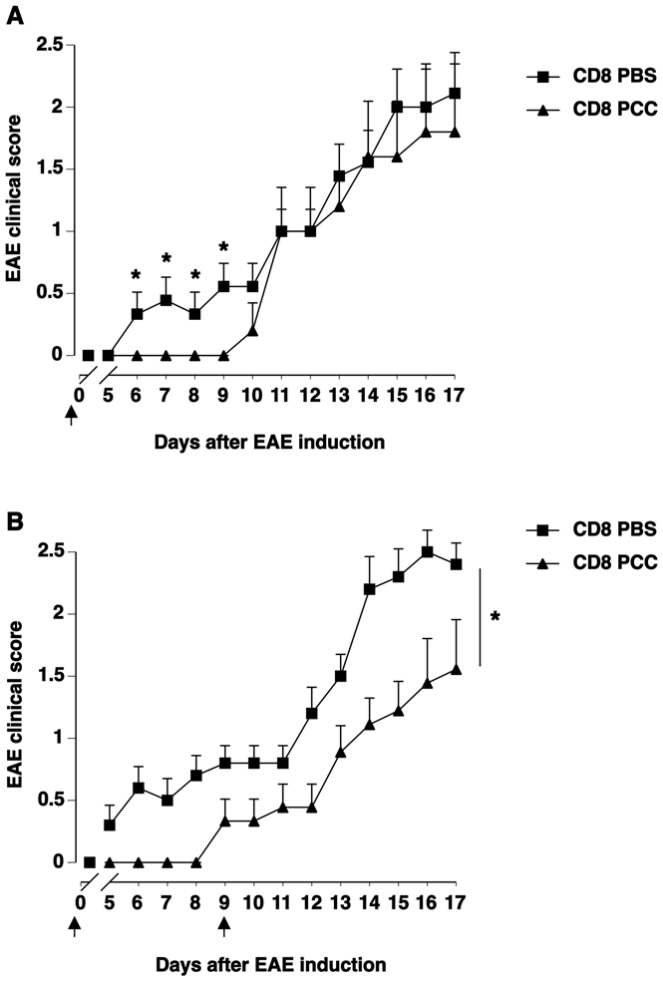
CD8+ T cells from PCC immunized mice protect naïve mice from EAE. A. Naïve C57BL/6 mice were immunized with PBS or 100µg of 50V PCC in CFA. Three weeks later, CD8+ T cells purified from the draining lymph nodes and spleen of the PBS immunized (CD8 PBS, squares) or PCC immunized (CD8 PCC, triangles) mice were adoptively transferred into naïve C57BL/6 recipients (CD8 PBS n = 10, CD8 PCC n = 10). Sixteen hours later, EAE was induced in the recipients upon subcutaneous immunization with MOG peptide accompanied by intravenous pertussis toxin on the day of EAE induction and 2 days later. B. Similar protocol was carried out with a second transfer of CD8+ T cells 9 days after EAE induction. The clinical course of EAE was analyzed using a paralysis grading score. Mean ± SEM. * indicates that the p-value<0.05 as assessed using ANOVA Fisher's PLSD. Arrows indicate the time points of CD8+ T cell transfer.

Vß-specific protection observed in our experimental set-up is akin to the protection induced upon injection of the vaccinating clones during TCV. Since CD8+ T cells possess an essential role in TCV-mediated protection [2], we questioned whether the Vß-specific protection observed in our model was also dependent on CD8+ T cells. To address this question, naïve mice were immunized with PBS or PCC. Three weeks later, CD8+ T cells purified from the draining lymph nodes and the spleen were adoptively transferred to naïve recipients. Sixteen hours later, EAE was actively induced in the recipients by immunization with MOG. Mice that received CD8+ T cells from PCC-immunized mice showed delayed signs of EAE development as compared to control mice that received CD8+ T cells from PBS-immunized mice (Figure 2A). The observation that the protection conferred by the CD8+ T cell transfer was of a lower magnitude than that observed in the initial experiments suggested that regulatory CD8+ T cells might need to be sustained to be effective. Indeed, the first experimental design where the two sequential immunizations were performed in the same mouse most likely favored the continuous generation or maintenance of regulatory CD8+ T cells due to the persistence of the first anti-PCC CD4+ T cell response. In an independent experiment, we therefore performed 2 consecutive adoptive transfers of CD8+ T cells: the first one was performed the day before EAE induction as in the initial experiment and the second, 7 days later. The development of EAE was further retarded in mice that received 2 injections of CD8+ T cells from PCC-immunized mice as compared to control mice (Figure 2B) suggesting that continuous Vß-specific CD8+ T cell generation or maintenance is required for the suppression of CD4+ T cell-mediated autoimmunity. The Vß-specific protection triggered by the first CD4+ T cell response is hence CD8+ T cell-dependent and is reminiscent of observations made in CD8+ T cell-deficient mice in which secondary T cell activation is unrestrained [8].

### Control of T cells by ‘naturally’-induced regulatory CD8+ T cells is Qa-1-dependent

**Figure 3:**
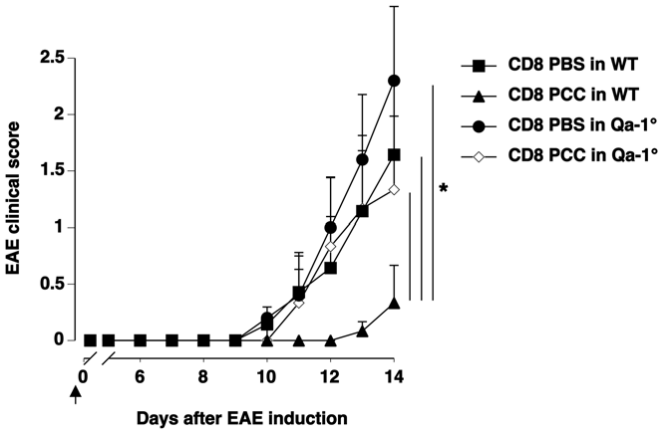
Regulatory CD8+ T cells are restricted to Qa-1. Naïve C57BL/6 mice were immunized with PBS or 100µg of 50V PCC in CFA. Three weeks later, CD8+ T cells purified from the draining lymph nodes and spleen of PBS-immunized (CD8 PBS) and PCC-immunized (CD8 PCC) mice were adoptively transferred into either wildtype (WT, n = 7 for ‘CD8 PBS in WT’ and ‘CD8 PCC in WT’) or Qa-1-deficient mice (Qa-1°, n = 5 for ‘CD8 PBS in Qa-1°’ and n = 6 for ‘CD8 PCC in Qa-1°’). EAE was induced in the recipients 16 hours later. * indicates that the p-value<0.05 as assessed using ANOVA Fisher's PLSD.

Our experiments illustrate that CD8+ T cells induced upon antigen-induced CD4+ T cell activation are specific for a particular Vß chain as the endogenous activation of CD4+Vß5+ T cells failed to confer protection against anti-MOG Vß8.2+CD4+ T cell-mediated EAE. Prior studies have demonstrated that peptides derived from the Vß chain are presented by Qa-1 on vaccinating T cells used during TCV [9], [10]. We investigated whether activated T cells specific for non-self antigens may also be capable of presenting Vß peptides in the Qa-1 molecule that would induce regulatory CD8+ T cells. CD8+ T cells from PCC- or control PBS-immunized mice were adoptively transferred to naïve Qa-1-deficient or wildtype mice. EAE development upon primary immunization with MOG in Qa-1-deficient mice has been demonstrated to follow disease kinetics comparable to wildtype mice [3]. As observed before (Figure 2), wildtype mice that received CD8+ T cells from PCC-immunized mice were protected during the preliminary stages of EAE development as compared to control mice (Figure 3). Interestingly, Qa-1-deficient mice that received CD8+ T cells from either PBS- or PCC-immunized donors were not protected from EAE development indicating that CD8+ T cell-mediated control required Qa-1 presentation on activated CD4+ T cells (Figure 3). These experiments indicate that CD4+ T cells induced upon pathophysiological antigen-induced activation are able to present Vß-peptides in their Qa-1 molecule thus enabling the generation of regulatory anti-Vß CD8+ T cells, which in turn recognize similar Qa-1-Vß peptide complexes on self-reactive CD4+ T cell and suppress their function.

### Regulatory CD8+ T cells diminish lymphocyte infiltration into the CNS

**Figure 4:**
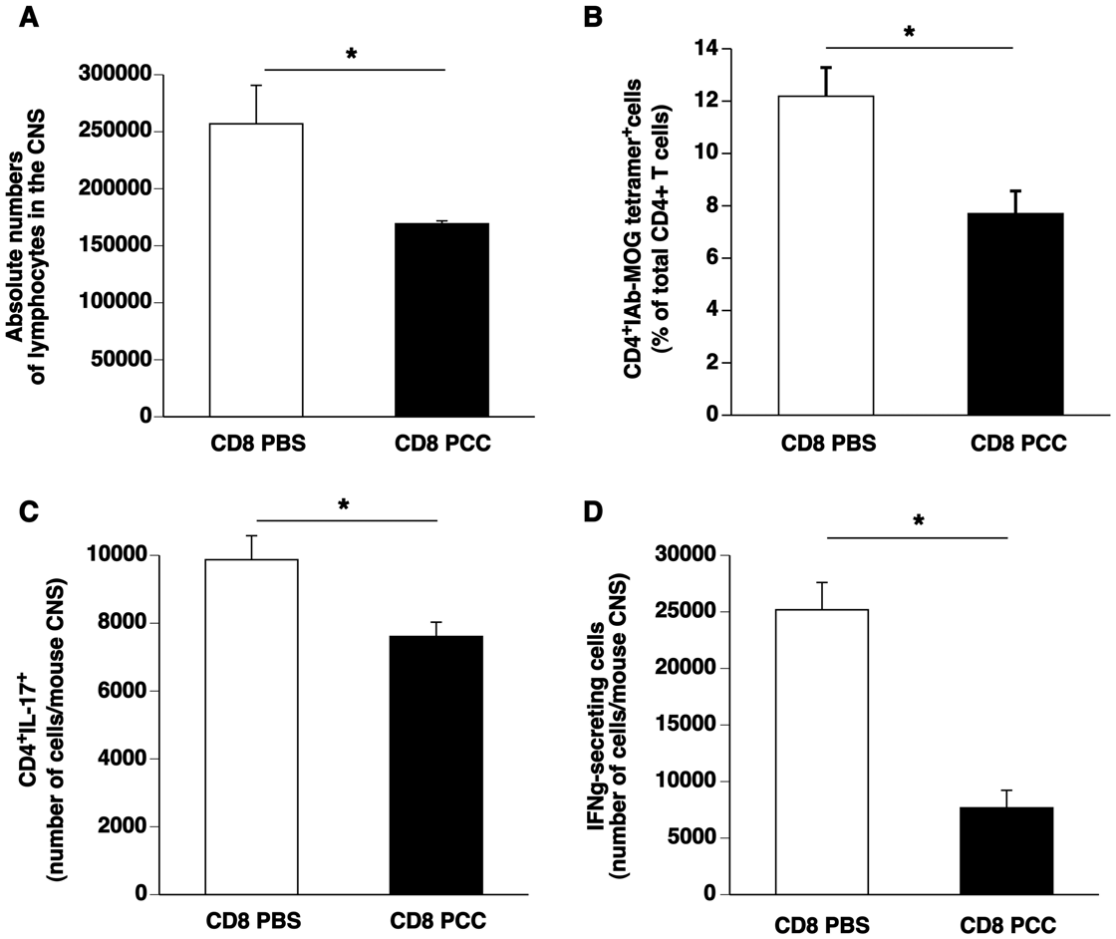
Regulatory CD8+ T cells prevent lymphocyte infiltration and CNS inflammation. Naïve mice were adoptively transferred with CD8+ T cells from PBS-immunized (CD8 PBS, n = 4) or PCC-immunized (CD8 PCC, n = 4) mice followed by EAE induction 16 hours later. Thirteen days after EAE induction, the mice were sacrificed and the lymphocyte populations in the brain and spinal cord were examined by flow cytometry. A. Absolute numbers of lymphocytes in the CNS gated according to their forward and side-scatter characteristics. B. MOG-specific CD4+ T cells were stained using MOG_38–49_-I-Ab tetramers. Represented is the percentage of tetramer-positive cells among total CD4+ T cells in the CNS. C. Absolute numbers of CD4+ T cells that produce IL-17 in the CNS. D. Absolute numbers of IFNγ-producing CD4+ T cells in the CNS. Mean ± SEM. * indicates that the p-value<0.05 as assessed using Mann-Whitney non-parametric analysis.

Active EAE development is associated with an infiltration of inflammatory lymphocytes in the central nervous system (CNS). The protection observed upon CD8+ T cell transfer from PCC-immunized mice was associated with a reduction in the number of lymphocytes in the CNS as compared to control mice (Figure 4A). Anti-MOG CD4+ T cells primed in the periphery migrate through the blood-brain barrier essentially by chemotaxis [11]. Once infiltrated these cells secrete pro-inflammatory cytokines such as IFNγ and IL-17 that trigger the inflammatory response facilitating myelin destruction. In our studies, the percentage of MOG-specific CD4+ T cells detected by tetramer staining was also diminished in the CNS of protected mice (Figure 4B). Correspondingly, in the CNS of protected mice we also observed a drastic diminution of IL-17-secreting CD4+ T cells (Figure 4C) and of IFNγ-secreting cells indicating the control of inflammation in the CNS of these mice (Figure 4D).

## Discussion

Regulatory Qa-1-restricted CD8+ T cells can be induced by the TCV - T cell vaccination -procedure that involves the injection of activated, attenuated, CD4+ T cells. The main result of the present study is the demonstration that similar regulatory cells can specifically be triggered upon the activation of endogenous T cells. Indeed, our data indicate that antigen-stimulated CD4+ T cells induce regulatory CD8+ T cells that recognize Vßx-derived peptides presented in the Qa-1 molecule on CD4+ T cells. Similar to TCV, regulatory CD8+ T cells could target CD4+ T cells with distinct antigenic specificity as long as they expressed a TCR with the same Vß chain. Indeed, secondary T cell responses involving T cells with Vß chains distinct from those activated during the primary response developed completely unrestricted, as demonstrated by our experiments performed with ovalbumin. In this case, the Qa-1-restricted CD8+ T cells elicited by anti-OVA Vß5 CD4+ T cells failed to control the encephalitogenicVß8.2 CD4+ T cells. In addition, the primary CD4+ T cell response occurred unhampered, indicating that regulatory CD8+ T cells do not affect primary immune responses. We propose that regulatory CD8+ T cells are induced at a later stage of the immune response during which, to the best of our knowledge, no regulation has yet been described. Such a system of regulation can be potentially crucial as T cell responses frequently entrail the release of a variety of T cell mediators that are not only efficient in the elimination of foreign antigens but can also destroy self tissue. After the essential primary response, the immediate re-initiation of inflammatory secondary responses could lead to drastic and permanent tissue damage. Induction of regulatory CD8+ T cells might hence limit tissue damage caused by constant re-activation of T cell responses when the specific antigen persists. Interestingly, studies on TCV-induced regulatory Vß-specific CD8+ T cells have shown that these cells are capable of specifically targeting inflammatory Th1 CD4+ T cells [9]. In our studies, we have shown that regulatory Qa-1-restricted CD8+ T cells are able to limit the secretion of IFN-γ from cells of the CNS and also to control CNS-infiltrating inflammatory IL-17-secreting CD4+ T cells, thus preventing tissue damage.

A corollary question concerns the site where Qa-1-restricted CD8+ T cells do exert their control over CD4+ T cells. The expression of Qa-1 on activated CD4+ T cells increases with time (unpublished personal observation). Vaccinating T cells used during TCV require stimulation for at least 40 hours before vaccination indicating a necessity for optimal surface expression of Qa-1-Vß peptide complexes [12]. Thus, for control by Vß-specific Qa-1-restricted regulatory CD8+ T cells, self-reactive CD4+ T cells need to be optimally activated. Given that activated CD4+ T cells exit reactive lymph nodes 72-96 hours after interaction with antigen presenting cells [13], we can assume that regulatory CD8+ T cells either control activated self-reactive T cells in the secondary lymphoid tissues or co-migrate to the target tissue (the CNS in our case) and control T cell function. The lack of tissue destruction upon transfer of protective CD8+ T cells together with the absence of inflammatory leukocytes in the CNS indicates that CD4+ T cells were probably not able to attain the CNS and cause inflammation. Such evidence suggests that regulatory CD8+ T cells control activated self-reactive CD4+ T cells in the reactive lymph node. In support of these findings, our recent data indicate that Qa-1-restricted anti-ergotypic CD8+ T cells are able to proliferate and control T cell responses in secondary lymphoid organs [14]. The recent finding by the group of Cantor [15] showing that Qa-1-restricted CD8+ T cells can control follicular helper T cells further indicates that the point of control of these regulatory cells is located in secondary lymphoid organs.

The fact that, in a physiopathological situation, regulatory CD8+ T cells could sequentially target CD4+ T cells with distinct antigen specificities, provided that they express a TCR with the same Vß chain, raises unforeseen questions. There exist 25 different Vß chains from which a mature T cell expresses one alone in its TCR. The establishment of a Vß specific regulatory system would thus allow the control of 1/25th of CD4+ T cells. One could argue that if these regulatory Qa-1-restricted CD8+ T cells become memory cells, secondary CD4+ T cell responses might be paralyzed after few primary CD4+ challenges, each with a distinct Vß chain. Since this is evidently not the case, we anticipate that the regulatory Qa-1-restricted CD8+ T cells are short-lived cells. Future studies are warranted to specifically address this issue.

Another important finding of our study is that Vßx-specific Qa-1-restricted CD8+ T cells induced upon CD4+ T cell responses against a non-self antigen were capable of controlling the activation of self-reactive Vßx+ T cells. Thus, our data point at a crucial role for CD4+ T cell responses against non-self antigens in the maintenance of an efficient peripheral regulatory Qa-1-restricted CD8+ T cell repertoire that can control the expansion of autoreactive T cells such as those involved in the EAE model.

**Figure 5:**
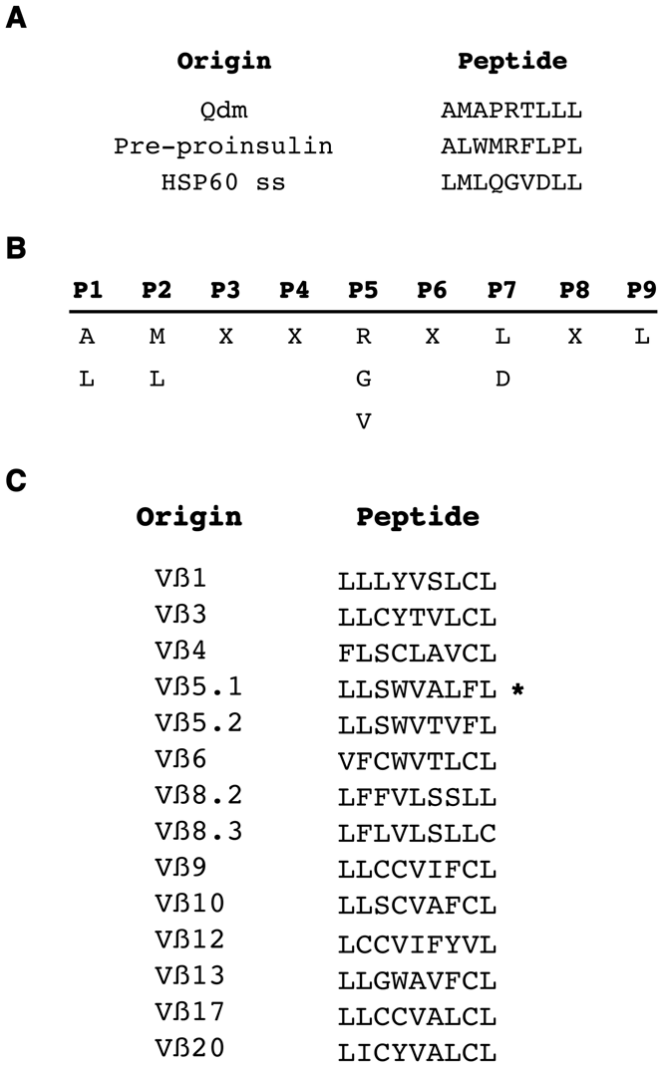
Qa-1-binding molecular pattern. A. The alignment of previously described 9 amino acid-long Qa-1-binding peptides (Qdm, pre-proinsulin, HSP60 signal sequence derived peptide) using the Geneious software (ClustalW analysis). B. A consensus sequence with conserved amino acids in position (P) 1, 2, 5, 7 and 9 was revealed from this alignement. C. The search of such a consensus sequence in mouse Vß chains derived from the IMGT database revealed the presence of consenting 9 amino acid long peptides in the leader sequences of all mouse Vß chains. * Described as being a Qa-1-binding peptide in ref [12].

In order to analyze the nature of the Vß sequences capable of binding Qa-1, we performed *in silico* alignments of the known repertoire of peptides (Figure 5) shown to bind Qa-1: peptides derived from the MHC class I-leader sequence [16], pre-proinsulin [17], [18] and Heat Shock Protein (HSP) 60 [19]. This revealed a consensus sequence (Figure 5B) that we searched for in complete mouse Vß chain sequences. We found candidate peptides in each Vß chain with this consensus pattern (Figure 5C). These Vß sequences not only possess the chemically and structurally defined consensus sequence of all known Qa-1 binding peptides but were also highly hydrophobic, a property common to MHC-leader sequences, making them ideally suited to bind Qa-1 that, like its human orthologue HLA-E, has a substantially hydrophobic peptide binding groove [20].

Most intriguingly, similar to the MHC/HLA leader sequence peptides, all the Vß peptides elucidated by sequence alignment mapped to the leader sequence of the TCR Vß chains (Figure 5C). Similar peptide sequences were also found in the leader sequences of human Vß chains (data not shown). Interestingly, these 9 aa-long sequences are considerably distinct from one another, an absolute requirement to allow Qa-1-restricted CD8+ T cells to distinguish each Vß peptide and to endow them with the capacity to specifically control CD4+ T cells according to their Vß chains. Indeed, Qa-1 and HLA-E might have evolved to preferentially bind peptides derived from leader sequences of membrane or secreted proteins. Upon recognition of their cognate peptide, T cells upregulate TCR expression, which requires the synthesis of new Vß chains, and hence, the release of free leader sequences. Thus, once in the MHC-peptide loading compartments, peptides derived from Vß leader sequences would compete with MHC leader sequences to bind Qa-1. Upon clonal T cell activation, the large number of T cells capable of expressing similar Qa-1-Vß peptide complexes would further favor the expansion of specific CD8+ T cells. However, recent studies have shown that Vß chain peptides that do not map to the leader sequence can induce a Qa-1-restricted CD8+ T cell response [21]. In these studies, Qa-1 was loaded with synthetic peptides from Vß CDR2 regions [21] or by cross-presentation of TCR-derived peptides upon processing of apoptotic CD4+ T cells by bone marrow-derived dendritic cells [22]. These data suggest that Qa-1-restricted CD8+ T cells can recognize distinct peptide repertoires according to the antigen-presenting cells. Our data suggest that in the case of activated CD4+ T cells, the Qa-1 molecule would preferentially present peptides derived from leader sequences of TCR Vß chains.

Our results demonstrate that a complementary, adapted, regulatory response is induced upon T cell responses against non-self antigens. We can anticipate that in individuals that mount few adaptive immune responses, due to infrequent antigen exposure, this type of regulation might be poorly developed thereby allowing the peripheral expansion of pathogenic self-reactive T cells. On the other hand, these regulatory CD8+ T cells could impair effective T cell responses during vaccination strategies. Further studies on the half life and mechanism of control of these regulatory CD8+ T cells would be fruitful to the understanding of differential responses of individuals to infections and vaccinations.
